# Well-being of family physicians during COVID-19 pandemic in Slovenia

**DOI:** 10.1186/s12875-024-02416-2

**Published:** 2024-05-31

**Authors:** Alina Verdnik Tajki, Špela Miroševič, Maja Cvetko Gomezelj, Ksenija Tušek Bunc, Esther Van Poel, Sara Willems, Zalika Klemenc-Ketiš

**Affiliations:** 1 Maribor Community Health Centre, Maribor, Slovenia; 2 Ljubljana Community Health Centre, Ljubljana, Slovenia; 3 Department of Family Medicine, University of Ljubljana, Ljubljana, Slovenia; 4 Piran Community Health Centre, Piran, Slovenia; 5 Department of Family Medicine, University of Maribor, Maribor, Slovenia; 6 Department of Public Health and Primary care, Ghent Univesity, Ghent, Belgium; 7 Quality and Safety Ghent, Department of Public Health and Primary Care, Ghent University, Ghent, Belgium

**Keywords:** Primary health care, Family physicians, Well-being, Multi-country, PRICOV-19, COVID-19, Quality of care, Risk factors, Mental health maintenance

## Abstract

**Background:**

During the COVID-19 pandemic, family physicians (FPs) served as the the initial point of contact for patients potentially infected with the virus, necessitating frequent updates to treatment protocols. However, practices also faced organizational challenges in providing care to other patients who also needed their medical attention. The pressure on FPs increased and affected their well-being. The international PRICOV-19 study, titled “Primary care in times of COVID-19 pandemic,” investigated how FPs functioned during the COVID‐19 pandemic. This article examines the correlation between various organizational and structural COVID-19-related variables and the well-being of FPs in Slovenia.

**Methods:**

Between October 2020 and January 2021, we conducted an online cross-sectional survey. The questionnaire was distributed to 1040 Slovenian FPs and 218 family medicine (FM) trainees. Part of the questionnaire assessed the cooperation and well-being of FPs. The Mayo Clinic Well-being Index was used for the assessment. FP’s well-being was also assessed descriptively by asking open-ended questions about maintaining mental health during the pandemic. Potential factors associated with FPs’ well-being were identified using a multivariate linear regression method.

**Results:**

The final sample comprised 191 participants (response rate 14.1%). The mean value ± standard deviation of the Mayo Well-being Index was 3.3 ± 2.6 points. The FPs with the poorest well-being had 5–15 years of work experience and worked in a practice where work could not be distributed in the absence of a co-worker without compromising the well-being of colleagues. Physical activity was identified as the most common method of maintaining mental health among FPs.

**Conclusions:**

The results of the study suggest that targeted interventions are needed to support FPs mid-career, increase resilience in practice, promote strong team dynamics, and prioritise physical activity in healthcare. Addressing these aspects can contribute to the well-being of individual FPs and the overall health of the healthcare workers.

**Supplementary Information:**

The online version contains supplementary material available at 10.1186/s12875-024-02416-2.

## Background

In March 2020, the World Health Organization officially declared the COVID-19 outbreak a pandemic [1, 2]. At that time, Slovenia had 1,230 active family physicians (FPs). By 2021, the density of FPs in Slovenia was 64 per 100,000 inhabitants, significantly lower than the European average of 105 FPs per 100,000 inhabitants [3]. Slovenia faces a significant shortage of FPs, which makes their work difficult even under typical circumstances. With the outbreak of the pandemic, the tasks and duties of FPs have increased even more, and their work has become mentally and physically demanding, which affected their well-being [4].

In Slovenia, there is a statutory health insurance system with a single public insurer that guarantees universal healthcare coverage. The primary health care (PHC) infrastructure is based on a network of community primary health care centers (CPHC) organized by municipalities and private physicians within the national primary health care system. The country hosts 61 CPHCs distributed across 10 administrative regions. Of these 3 CPHCs have more than 50 family medicine teams, while about a third of the CPHCs are smaller and consist of up to five teams. Overall, Slovenia has about 900 PHC teams, whith about 200 operating as private facilities with concessions. In the midst of the pandemic, the reorganization of primary care became crucial to effectively treat suspected cases of COVID-19, continue care for other patients, including those with chronic conditions, and manage the impact of the health crisis [5, 6].

Throughout the pandemic, the Ministry of Health (MH) assumed responsibility for establishing national medical care priorities, formulating regulations and guidelines, and coordinating the procurement of essential equipment [7]. Simoltaneously, the Slovenian Medical Chamber (SMC) organized training courses and disseminated crucial professional and organizational information.

Due to the lack of standardised treatment protocols for COVID-19 patients and the simultaneous treatment of other patients, especially at the beginning of the pandemic, numerous logistical problems also arose at the secondary level. This placed an additional burden on the FPs. Furthermore, the diagnosis and treatment of chronic patients, typically managed in specialised hospitals at the secondary or tertiary care levels, were transfered to the already overstretched primary care level, compounding the workload.

In addition, FPs were forced to restrict the number of patient visits in order to implement the measures to contain the spread of the new coronavirus. This led to problems with the accessibility of FPs, significantly affected older people who are not as familiar with the information technology used to communicate with FPs remotely. Studies indicate that the number of in-person visits to physicians decreased by 25% during the pandemic [8].

The rapid and extensive spread of COVID-19 was unprecedented in recent history, placing a significant strain on the healthcare system. Moreover, until December 2020, there were no approved drugs or vaccines against the virus in Slovenia, which heightened the daily risk of illness and death for frontline care providers.

Since a pandemic of this scale is unprecedented, there is minimal understanding of its effects on the mental well-being of healthcare workers [9]. Preliminary data from the COVID-19 experience in China indicate that healthcare workers directly engaged with COVID-19 patients exhibited significantly higher levels of fear, anxiety, and depression compared to their colleagues in less vulnerable areas. In addition, all healthcare professionals experienced significantly higher levels of fear, anxiety and depression than other non-medical hospital staff [10].

Several studies demonstrated that during the pandemic, a majority of FPs reported elevated stress levels, with the incidence being twice as high among women compared to men [11]. The following factors contributed to stress: difficult decision making, fear of patients, information overload, high workload, feeling alone, and feeling that working hours interfered with personal life.

Factors associated with less stress were: Consistency at work, feeling useful, having confidence in the future and being able to forget about work at home. Overall, respondents felt supported by their departments and facility and felt that emergency plans and personal protective equipment were adequate [9, 11].

There is limited understanding about the relationship between practice organization and potential risk factors for stress in FPs. Oru study aimed to explore relationship between various organizational and structural factors and the well-being of FPs during the COVID-19 pandemic.

## Methods

### Study design and settings

This study was part of a large-scale international cross-sectional study PRICOV-19, which was conducted in primary health care facilities in 37 European countries and Israel. The study was initiated by Quality and Safety Ghent (Q&S Ghent), an interdisciplinary competence centre for quality and safety in primary and transmural care at the University of Ghent (Belgium). As part of this study, an international research consortium was formed involving more than 45 universities and research institutes from 38 countries, including the Department of Family Medicine of the Faculty of Medicine of the University of Ljubljana in collaboration with the Department of Family Medicine of the Faculty of Medicine of the University of Maribor. The PRICOV-19 study examines the organizational structures of family medicine practices in 38 countries in the midst of the COVID-19 pandemic, focusing on ensuring safe, effective, patient-centred and equitable care. The research covers the changes in roles and responsibilities in these practices, as well as staff well-being. In addition, PRICOV-19 aims to analyse relationships with the characteristics of both the practices and the healthcare system in general [1].

### Participants

We conducted an online cross-sectional survey targeting all FPs registered in Slovenia. A prerequisite for participation was that the FPs were actively working in family practice and consented to data collection and analysis. Additionally, we included FM trainees, who are physicians in the midst of their four-year family medicine specialisation. We excluded general FPs who were not actively practsing as FPs in Slovenia. Therefore, the invitation to participate was sent to 1040 Slovenian FPs and 218 FM trainees.

### Data collection

In order to reach the participants, we distributed the survey link through the official mailing list of the Network of Medical Professionals of the Medical Chamber of Slovenia. Participants were requested to complete an online self-report questionnaire via the REDCap (Research Electronic Data Capture) platform [12]. According to the study protocol, the questionnaire could only be completed by one FP or FM trainee per practice. Data collection concluded within four months, by January 2021 [1].

### Measurements

The questionnaire used was developed and validated at the University of Ghent and translated into Slovenian using the forward-backward method, which was the same in all 38 participating countries [1]. The questionnaire consisted of 53 questions divided into six sections: Patient flow (including appointment scheduling, triage and safety management for routine primary health care), infection prevention, information handling, communication with patients, collaboration, collegiality and self-care, and finally characteristics of participants and FP practice [1]. The final section of the survey included the Mayo Clinic Well-being Index [13], a validated instrument that allows individuals to assess their well-being in comparison to their professional colleagues. This index comprises nine questions covering emotional exhaustion, depersonalization, depression, fatigue, stress, and both mental and physical quality of life. After answering the nine questions of the index, respondents receive their individual score instantly. Scores on the index range from − 2 (indicating the lowest risk of stress) to 9 (indicating the highest risk of stress). A score exceeding 2 is regarded as indicative of mental stress risk.

### Statistical analysis

The data were analysed using SPSS Statistics for Windows Version 27.0 (IBM Corp., Armonk, N.Y., USA).

All patients were included the initial presentation of sample characteristics. However, for the main linear regression analysis, respondents with missing data on the primary outcome (Mayo Clinic Well-being Index) were excluded. The results of the Mayo Clinic Well-being Index questionnaire and missing values were treated according to the Mayo Clinic Well-being Index manual [14]. The primary outcome was FP well-being. The threshold provides a way to estimate the risk for distress in a group of FP that meet or exceed a specified value. FPs above the specified value are at higher risk of distress, which may leed to personal or professional consequences.

Various potential factors associated with FPs’ well-being in Slovenia where included in the analysis such as: years of work experience, FPs vs. FM trainees, size of practice, members working in the practice, location of practice (in terms of degree of rurality), availability of medical equipment needed in the context of COVID-19, ability to fill in the absence of FP, frequency of staff meetings in the practice, patient and staff safety measures, and contact with patients. Patient and staff safety measures were scored with a maximum of 9 points (triage, waiting room, infection control practices, structural changes in the reception area, telephone triage, video consultations, prescription repeat procedures and use of mail). Univariate analysis was performed for all variables, with those achieving statistical significance at the 0.2 level subsequently subjected to further analysis in a linear regression model using the Enter method. The final model aimed to identify factors associated with FP well-being, with variables achieving significance at *P* < 0.05. In the last part, the FPs also answered an open-ended question on how they maintain their well-being during a pandemic, which was empirically analysed by thematic analysis.

## Results

### Sample characteristics

The final sample consisted of 191 participants, resulting in a response rate of 14.1%. In the descriptive analysis, 132 participants provided written response to the open-ended question “In what ways do you maintain your mental health?“.

Seventy-eight (40.8%) of respondents had between 0 and 5 years of work experience, 40 (20.9%) between 5 and 15 years and 38 (19.9%) between 15 and 25 years. Thirty-five (18.4%) had more than 25 years of work experience. The average practice size was 1752 (± 604.8) patients. There was only 1 (0.5%) practice with three FM trainees, 12 (6.5%) with two FM trainees and 54 (29%) with one trainee. Additionally 119 (64%) practices had no trainees (see Table 1). On average 3.3 (± 1.1) paid staff members working in each practice with 0.8 (± 1.0) staff unable to work due to COVID-19 infection. The safety measures for patients and staff were followed by an average of 4.4 (± 1.2).

Eighty-two (43.2%) of the GP practices were located in large city centres. 65 (34.2%) were located in suburbs/small towns, 26 (13.7%) had mixed locations and 17 (8.9%) were located in rural areas. At the time of the study, COVID-19 protective equipment was available in 119 (71.3%) GP practices. Further characteristics of the sample and practices are detailed in Table 1.

**Table 1:** Description of the sample and its characteristics

**General information**	***N*** **(%)**
Position in the practice (*n* = 191)	
Family physician	154 (80.6)
Family medicine trainee	37 (19.4)
Work experiences (*n* = 191)	
0 to 5 years	78 (40.8)
5 to 15 years	40 (20.9)
15 to 25 years	38 (19.9)
25 years and over	35 (18.4)
Number of family medicine trainees in each general practice (*n* = 186)	
None	119 (64.0)
One	54 (29.0)
Two	12 (6.5)
Three	1 (0.5)
Location of general practice (*n* = 190)	
Big inner city	82 (43.2)
Suburbs	65 (34.2)
Mixed (urban-rural)	26 (13.7)
Rural	17 (8.9)
Equipment availability (*n* = 167)	
Yes No	119 (71.3) 48 (28.7)
**Team participation**	**Frequency (%)**
If staff member is absent due to COVID-19, work can be organized in a way that well-being of the colleagues is not compromised. (*n* = 159)	
Strongly disagree	15 (9.4)
Disagree	58 (36.5)
Neutral	20 (12.6)
Agree	55 (34.6)
Strongly agree	11 (6.9)
If staff member is absent due to COVID-19, practice can count on other FPs to help. (*n* = 163)	
Strongly disagree	2 (1.2)
Disagree	18 (11.0)
Neutral	13 (8.0)
Agree	78 (47.9)
Strongly agree	52 (31.9)
COVID-19 pandemic has promoted cooperation between FPs. (*n* = 159)	
Strongly disagree and disagree	29 (18.2)
Neutral	39 (24.5)
Agree	70 (44.0)
Strongly agree	21 (13.3)
How often is a meeting planned to discuss directives? (*n* = 160)	
Never	11 (6.9)
Rarely	12 (7.5)
Sometimes	36 (22.5)
Regularly	67 (41.9)
Always	34 (21.2)

### Well-being of family physicians during COVID-19 in Slovenia

The mean ± standard deviation of the Mayo Well-being Index was 3.3 ± 2.6 points (Table 2). The minimum value was − 2 and the maximum value was 8.

**Table 2:** Mayo index well-being evaluation

Well-being index evaluation (*n* = 163)	Frequency (%)
Emotional exhaustion	
No	60 (36.8)
Yes	103 (63.2)
Depersonalisation	
No	70 (42.9)
Yes	93 (57.1)
Depression	
No	89 (54.6)
Yes	74 (45.4)
Fatigue	
No	157 (96.3)
Yes	6 (3.7)
Stress	
No	73 (44.8)
Yes	90 (55.2)
Effect on mental quality of life	
No	59 (36.4)
Yes	103 (63.6)
Effect on physical quality of life	
No	108 (66.3)
Yes	55 (33.7)
The work I do is important to me (before COVID-19)	
Strongly disagree	0 (0)
Disagree	0 (0)
Slightly disagree	1 (0.6)
Neutral	11 (6.8)
Slightly agree	23 (14.1)
Agree	39 (23.9)
Strongly agree	89 (54.6)
The work I do is important to me (since COVID-19 started)	
Strongly disagree	13 (8.0)
Disagree	7 (4.3)
Slightly disagree	18 (11.0)
Neutral	39 (23.9)
Slightly agree	23 (14.1)
Agree	33 (20.2)
Strongly agree	30 (18.5)

### Analysis of potential factors for well-being

Analysing the potential factors related to well-being, only the impact on FP well-being due to the absence of infected staff and the frequency of policy discussion meetings were found to be statistically significantly related to FP well-being as assessed by the Mayo Well-Being Index. However, work experience (specifically, those with 5 to 15 years of work experience) was also included in the multivariate regression model due to the pre-specified threshold of *p* < 0.02 (see Methods). Further details of the univariate analysis results can be found in Appendix 1.

### Multivariate regression model of factors related to the pandemic and well-being

Multicollinearity was thoroughly checked before starting the multivariate analysis, and the variance inflation factor (VIF) was well below the threshold of 10. This result indicates that there are no significant multicollinearity issues in the data. In the multivariate regression model (Enter method), we found that the FPs who feel worst have 5–15 years of experience and work in a practice where, in the absence of a colleague due to COVID-19, work cannot be distributed in a way that does not affect the well-being of colleagues when controlling for other variables included in the analysis (Table 3). The model was found to be statistically significant (*p* < 0.001) and explained 13.6% of the variance.

**Table 3:** Final model of the linear multivariate regression analysis with significant predictors of the Mayo Well-being Index

Variable	B	95% Cl	β	t	*P*-value
Size of the practice (ref. > 25 years)					
0–5 years	0.552	-0.514, 1.618	0.106	1.023	0.308
5–15 years	1.372	0.140, 2.618	0.225	2.201	0.029
15–25 years	0.094	-1.115, 1.303	0.015	0.154	0.878
Location of general practice (ref. big inner city)					
Suburbs	0.210	-0.657, 1.077	0.039	0.478	0.633
Mixed (urban-rural)	0.079	-1.156, 1.314	0.011	0.127	0.899
Rural	0.951	-0.415, 2.317	0.112	1.375	0.171
Impact on well-being of FPs due to absence of infected staff members	-0.627	-0.956, -0.299	0.292	-3.771	< 0.001
Frequency of meetings to discuss directives since COVID-19	-0.182	-0.556, 0.192	-0.079	-0.962	0.338
Number of FPs and family medicine trainees working in the practice	-0.248	-0.561, 0.065	0.121	-1.567	0.119

*R* = 0.431; *R*^2^ = 0.186; adjusted *R*^2^ = 0.136; SE of the estimate = 2.345; B is unstandardized regression coefficient with 95% Confidence Interval; β is standardized regression coefficient

### Maintaining mental health

One hundred thirty-two FPs provided a written response to the open-ended question, *“*In what ways do you maintain your mental health*?*“ Six themes emerged from these comments: (1) physical activities; (2) hobbies; (3) feeling supported; (4) expressing emotions to other people; (5) spiritual support; and (6) unable to answer (see Table 4).

**Table 4:** Identified themes from the participants’ comments with representative quotes

Theme	Frequency (%)	Answers
Physical activities	104(78.8)	“I walk.“, “I run.“, “I am physically active 3 times per week at least 30 minutes.“, “I dance.“, “I work-out.”
Hobbies	56(42.4)	“I read books.“, “I watch TV, films, comedies.“, “I bake.“, “I listen to the music.“, “I climb and I clean in free time.“, “I do art and different hand crafts.“, “I learn how to play a flute and a piano.”
Feelings of being supported	51(38.6)	“I spend every possible minute with my family.“, “I spend time with my child, what makes me feel happy and important.“, “I spend time with my children and husband.“, “I am really happy when somebody notice my work and give me a compliment.”
Expressing emotions to other people	32 (24.2)	“I talk with my friends, family members, and my partner.“, “I talk with my colleagues at work.“, “I make videoconference with my family members to talk with them.”
Spiritual support	14 (10.6)	“I pray a lot.“, “I meditate.“, “I’ve got faith.”
Miscellaneus	4 (3.0)	“It is difficult for me to do anything, I don´t have enough time. I am not satisfied with my mental health. I can´t find enough time to do sport, which was verry important to me.“, “I don’t know the way yet.“, “It is difficult to do anything.“, “I wait for retirement in about a year. And I wait for appointment at specialist.”

The first theme has shown revealed that the best way for FPs to maintain their mental health is physical activity. Most FPs are regularly physically active, most of them go walking, running and dancing. Some of them also go hiking, rock climbing, do yoga and Pilates.

The second theme of the responses defined hobbies such as reading books, watching TV, listening to music and playing instruments as an important means of maintaining mental health before and during COVID-19.

The third theme reflected the FPs’ feelings of being supported by their families, colleagues, friends and team members.

The fourth theme of responses related to expressing feelings to other people. The FPs expressed their feelings and emotions mainly in conversations. Three of them also mentioned psychotherapists and relaxation techniques.

The fifth theme included respondents who thought that religion and spirituality are a good way to cope with daily stress.

The sixth theme represents a group of FPs who do not know how to maintain their mental health.

## Discussion

Slovenian FPs faced a high risk of stress exhibited poorer well-being during the COVID-19 pandemic. Notably, FPs with 5 to 15 years of work experience reported lower well-being levels. Poorer well-being was also reported in practices where staff were absent due to infections.

There is not much research on the well-being of physicians during the pandemic, but the results of existing studies are consistent and point to burnout and increased work-related stress [11, 15–17]. The mean well-being index in our study was 3.3 points, surpassing the threshold for distress risk. A total of 111 (68.5%) were identified as being at high risk and experiencing poor well-being. In the midst of the pandemic, primary health care workers’ professional obligations interfered with their personal lives significantly more than the reciprocal impact of personal matters on their professional lives. At the same time, the positive influence of work life on private life and vice versa was less pronounced compared to the interference. These circumstances correlated with an increased perception of stress among primary healthcare workers [18]. The lack of personal contact and less synchronised communication also had a negative impact on teamwork and morale among primary care provider staff during the pandemic [19].

Even prior to the pandemic, Slovenian FPs were confronted with numerous organizational problems at the primary level. A shortage of physicians, an excessive daily flow of patients and a high administrative burden are part of the daily work of FPs [20]. The COVID-19 pandemic has further aggravated the situation by causing excessive organizational stress [21].

Organizational adaptations at the primary level became imperative during the pandemic were needed immediately. The state changed the guidelines for the treatment of COVID-19 patients and other regulations on a daily basis. FPs were forced to monitor and follow the guidelines and apply them in daily practice, which also entailed more demanding working conditions [21, 22].

The need to follow instructions and rules that are perceived as inadequate, the lack of resources to meet patients’ needs, excessive numbers of patients and poor personal relationships between staff, worn-out practice software were cited as the most serious stress factors [23, 24]. The results of our study show that physicians with fewer years of work experience report poorer well-being, which has also been shown in other studies [25–27]. This finding is probably a consequence of the impact of work on the private and family life of physicians, in particular a conflict between the professional role and the role as a parent [23].

There is an urgent need to implement systematic preventive measures in the area of mental health for primary healthcare care workers. It is crucial to design and implement programs aimed at promoting resilience, which is an important protective factor in times of mental stress.

During the pandemic, there has been a notable rise in sickness absence among healthcare workers. Literature indicates that this increase is attributed to healthcare workers becoming infected with virus and/or wider sector-wide impacts, such as strict infection control measures [28, 29].

The ongoing COVID-19 pandemic has profoundly reshaped the dynamics of primary care work, communication, and learning [30]. All these issues, combined with the increased sick leave of healthcare workers, can effect on the well-being of the healthcare workers, as detected in our study [31]. To be able to cope with the stress, healthcare workers must develop and implement different strategies [31]. In our study, physical activity emerged as the most crucial method for maintaining stress during the pandemic. Physical plays a vital role in stress reduction and contributes to overall well-being [32].

Various hobbies, feeling supported, expressing emotions to other people and spiritual support were also high on the list of responses. Spending time with family and talking about problems were the most common responses. Studies suggest that spending time with family during leisure time can play a positive role in maintaining mental health [33].

On the contrary, our study also identified FPs who lacked strategies to cope with stressful situations or had lost motivation to enhance their well-being. Some of them sought help from psychotherapists. In a study on young doctors’ anxiety about their professional future, almost all participating doctors agreed that some stress management techniques should be taught during their studies. It should be borne in mind that young doctors are generally not fully aware of the future role of their coping resources [23].

The strength of our study is the use of a validated international questionnaire [1]. In Slovenia, the questionnaire was sent to all active FPs using the official mailing list of the Slovenian Medical Chamber.

One of the limitations was that the questionnaire was very long and therefore the number of completed forms was quite low compared to the number of all active FPs, resulting in a low number of participants. Due to the response rate and the lack of information on the characteristics of non-respondents, it is not possible to generalize the results to the population as a whole.

The data collection took place from October 2020 to January 2021 and covered several phases of the pandemic, which were characterized by waves and quieter phases since its outbreak in March 2020. The organizational and psychosocial landscape during this period showed significant differences, which may have had an impact on the burden on professionals. The temporal aspect is crucial in the area of well-being at work, as the conditions experienced by individuals can differ significantly depending on the specific phase of the pandemic. Therefore, examining the associations between current psychological and physical distress and organizational and psychosocial distress, which were subject to variation over an eight-month period, may limit the relevance of the findings.To assess well-being, we used the Mayo Clinic Well-being index. This tool has been developed to provide healthcare providers with an immediate response to their physical and mental state and also to use in research [34]. As evident from the recent systematic review, there are 8 instruments for measuring psychological distress [35]. This review found that due to the small number of included studies per instrument and the poor quality of the included studies, it is not clear whether the diagnostic accuracy of instruments to screen for psychological distress is sufficient. This lack of information makes it difficult to select the “best” instrument for screening for psychological distress and the risk of psychological distress. However, the few available measures in the studies were mostly sufficient, including the Mayo Well-Being Index.

## Conclusion

During the COVID-19 pandemic, the well-being of Slovenian FPs, particularly younger ones, was notably compromised. Practices with more absenteeism due to healthcare worker infections were more at risk.

While our study revealed that FPs employed various methods to cope with stress, the effectiveness of these strategies remains uncertain.

The results of the study suggest that targeted interventions are needed to support mid-career FPs, increase practice resilience, promote strong team dynamics and prioritize physical activity in healthcare settings. Addressing these aspects can contribute to the well-being of individual FPs and the overall health of healthcare workers.

In anticipation of future healthcare emergencies, it is imperative that all primary care professionals are trained in the techniques to effectively deal with increased stressors. These interventions should be evidence-based and available online in the form of asynchronous learning.

### Supplementary Information

Supplementary Material 1

## Data Availability

All data are centrally stored on the Ghent University server (Belgium). All data is pseudomised at Ghent University, and all raw data that could lead to the identification of the participants is permanently removed. As a researchers from partnering institutions we are able to access non-identifiable data from their national database after data cleaning [1].

## Abbreviations

FP: Family physician
FM: Family medicine
PRICOV-19: Quality and safety in PRImary care in times of COVid-19
