# Supplementary material for: Well-being of family physicians during COVID-19 pandemic in Slovenia
Source: BMC Prim Care. 2024 May 31;24(Suppl 1):289. doi: 10.1186/s12875-024-02416-2 (PMC11143559; doi:10.1186/s12875-024-02416-2)
Supplement: Supplementary file 1 — Supplementary Material 1 [file 12875_2024_2416_MOESM1_ESM.docx]

Appendix 1: Univariate analysis of all potential factors associated with the FPs well-being

| **Variable** | **F/t value** | **P-value** |
| --- | --- | --- |
| Size of the practice (Pearson Correlation, 2-tailed) |  | 0.401 |
| Years of work experiences (ref. 0-5 years) (ANOVA)  5-15 years  15-25 years  25 years and over | 6.80 | 0.010 |
| Staff working in the practice (Pearson Correlation, 2-tailed) |  | 0.385 |
| General practice with versus without family medicine trainee (ANOVA) | 0.046 | 0.830 |
| Location of the general practice (ref. big inner city) (ANOVA)  Suburbs  Mixed (urban-rural)  Rural | 1.80 | 0.194  0.184 |
| Absent of staff members due to COVID-19 infection (Pearson Correlation, 2-tailed) |  | 0.484 |
| Equipment availability (Pearson Correlation, 2-tailed) |  | 0.385 |
| Impact on the well-being of FPs due to absence of infected staff members (Pearson Correlation, 2-tailed) |  | <0.001 |
| Help of another FP in case of absence of staff members in general practice (Pearson Correlation, 2-tailed) |  | 0.249 |
| The impact of COVID-19 on increased cooperation between FPs (Pearson Correlation, 2-tailed) |  | 0.745 |
| Frequency of meetings to discuss directives (Pearson Correlation, 2-tailed) |  | <0.001 |
| Patient and staff safety measure (Pearson Correlation, 2-tailed) |  | 0.742 |
